# From BEST1 mutations to retinal regeneration: integrating stem cell–derived RPE models and gene correction strategies

**DOI:** 10.3389/fcell.2026.1832772

**Published:** 2026-05-15

**Authors:** Yan Wang, Sihua Cheng, Yu Zhang, Tingting Yang

**Affiliations:** Department of Ophthalmology, Columbia University, New York, NY, United States

**Keywords:** bestrophin-1(BEST1), calcium-activated chloride channel, gene therapy, hiPSC-derived RPE, retinal pigment epithelium, retinal regeneration, stem cells, bestrophinopathy

## Abstract

Retinal degenerative diseases are among the leading causes of irreversible vision loss worldwide and arise primarily from progressive dysfunction and death of photoreceptors and retinal pigment epithelial (RPE) cells. Because the mammalian retina lacks an intrinsic capacity for regeneration, current treatments remain limited and largely palliative. Recent advances in stem cell technologies and gene-based therapies, however, have opened new avenues for retinal repair and functional restoration. Among monogenic retinal disorders, BEST1-associated retinopathies provide a particularly informative paradigm for linking molecular mechanisms to emerging regenerative strategies.The human BEST1 encodes bestrophin-1 (BEST1), a calcium-activated chloride channel predominantly expressed in the RPE, where it plays essential roles in ionic homeostasis, transepithelial transport, and regulation of the visual cycle. Pathogenic variants in BEST1 give rise to a spectrum of inherited retinal diseases, including Best vitelliform macular dystrophy, autosomal recessive bestrophinopathy, and adult-onset vitelliform dystrophy. Mechanistic studies of BEST1 mutations have revealed diverse functional consequences, ranging from loss-of-function to gain-of-function effects, highlighting the importance of precise molecular diagnosis for therapeutic intervention.Here, we synthesize recent progress in stem cell–derived RPE models and gene correction strategies, using BEST1-associated retinopathies as a conceptual framework. We discuss how human induced pluripotent stem cell–derived RPE systems enable disease modeling and functional analysis of pathogenic variants, and how gene replacement and genome editing approaches are tailored to distinct mutation classes. Finally, we explore how integration of stem cell and gene therapy strategies may advance retinal regeneration and outline future directions for personalized and mechanism-based treatments of retinal degenerative diseases.

## 1 Introduction

Retinal degenerative diseases, including age-related macular degeneration (AMD) and inherited macular dystrophies, represent a major cause of permanent vision loss and blindness. These disorders are characterized by progressive degeneration of photoreceptors and retinal pigment epithelial (RPE) cells, leading to disruption of the visual cycle and irreversible impairment of visual function. The RPE is a highly specialized epithelial monolayer that performs essential supportive roles for photoreceptors, including nutrient transport, phagocytosis of photoreceptor outer segments, ion and water homeostasis, and regulation of the retinoid cycle.

A fundamental challenge in treating retinal degenerative diseases is the limited regenerative capacity of the mammalian retina. Unlike lower vertebrates, mammalian retinal neurons and RPE cells do not undergo spontaneous regeneration following injury or degeneration. Consequently, conventional pharmacological approaches have had limited success in restoring lost vision once cellular degeneration has occurred. These limitations have driven intense interest in regenerative strategies based on stem cells and gene therapy.

Stem cell–based approaches, including those using embryonic stem cells (ESCs), induced pluripotent stem cells (iPSCs), mesenchymal stem cells (MSCs), and retinal progenitor cells (RPCs), aim to replace or support degenerated retinal cells and restore tissue function. In parallel, gene therapy strategies seek to correct or compensate for pathogenic mutations underlying inherited retinal diseases. Recent advances in viral vector engineering and genome editing technologies have substantially improved the feasibility of these approaches.

In this review, we focus on BEST1-associated retinopathies as a model system for understanding how mechanistic insights into ion channel dysfunction can inform regenerative medicine. *BEST1* mutations provide a unique opportunity to integrate molecular studies of channel function with stem cell–derived disease models and gene correction strategies. By examining these advances in a unified framework, we highlight how mechanistic understanding can guide therapeutic innovation and contribute to broader efforts in retinal regeneration.

## 2 BEST1 and retinal health

### 2.1 Overview of BEST1 and the bestrophin family

Bestrophins are widely recognized as calcium-activated chloride channels (CaCCs) that form pentameric assemblies in the plasma membrane. *BEST1* encodes BEST1, the founding member of the bestrophin family of proteins, which also includes BEST2, BEST3, and BEST4 in humans. Among family members, BEST1 is predominantly expressed in the basolateral membrane of RPE cells, whereas other paralogs show broader expression patterns in epithelial and neural tissues (Hartzell et al., 2005; Strauss, 2005; Johnson et al., 2013; Li Y. et al., 2017; Ji et al., 2019b; Owji et al., 2021; Zhao et al., 2021).

Structurally, bestrophins share a conserved N-terminal transmembrane region responsible for pore formation and ion conduction, and a cytoplasmic C-terminal domain involved in channel regulation and protein–protein interactions (Yang et al., 2014b; Owji et al., 2020; Owji et al., 2021; Owji et al., 2022a; Wang et al., 2024; Phan et al., 2025). Functional studies have demonstrated that bestrophins mediate calcium-dependent chloride currents and contribute to epithelial ion transport and volume regulation (Yang et al., 2015; Owji et al., 2022a). In the RPE, these channels are thought to participate in transepithelial chloride flux, coupling ionic transport to fluid movement and metabolic signaling pathways (Zhang et al., 2018; Owji et al., 2022b; Owji et al., 2024; Wang et al., 2024).

### 2.2 Physiological roles of CaCCs in RPE

The RPE plays a central role in maintaining retinal homeostasis by regulating ionic composition in the subretinal space, controlling water transport, and supporting photoreceptor metabolism. CaCCs, including BEST1, are key components of this regulatory network (Esumi et al., 2009). By responding to intracellular calcium signals, these channels modulate chloride conductance and influence membrane potential, thereby coordinating ion transport with cellular activity.

BEST1 has been implicated in generating the electro-oculogram “light peak,” a clinical measure of RPE function, and in maintaining appropriate ionic gradients across the RPE layer (Marmorstein et al., 2006). Disruption of BEST1 function alters transepithelial transport properties and may impair the RPE’s ability to support photoreceptor survival. Beyond its role in ion conduction, BEST1 has also been linked to interactions with metabolic enzymes and signaling proteins, suggesting that it integrates ionic and metabolic regulation within the RPE (Zhang et al., 2018; Owji et al., 2024; Wang et al., 2024).

### 2.3 Mutation types and pathogenic mechanisms

More than 250 mutations in the *BEST1* gene have been identified and are associated with a spectrum of retinal degenerative disorders collectively termed bestrophinopathies (Johnson et al., 2014; Yang et al., 2014b; Guziewicz et al., 2016; Johnson et al., 2017; Sinha et al., 2020). These include Best vitelliform macular dystrophy (BVMD), autosomal recessive bestrophinopathy (ARB), adult-onset vitelliform macular dystrophy (AVMD), and autosomal dominant vitreoretinochoroidopathy (ADVIRC) (Marmorstein et al., 2000; Marmorstein and Kinnick, 2007; Marmorstein et al., 2015; Yang et al., 2015; Carter et al., 2016; Johnson et al., 2017; Kolesnikova et al., 2022). And in some reports, few of them are associating with a diagnosis of retinitis pigmentosa (RP) (Davidson et al., 2009; Dalvin et al., 2016; Johnson et al., 2017). Clinical phenotypes vary widely in age of onset, severity, and pattern of retinal involvement, reflecting diverse molecular consequences of different mutations.

Mechanistically, *BEST1* mutations can be broadly categorized into loss-of-function and gain-of-function variants (Ji et al., 2019a; Ji et al., 2019b; Zhao et al., 2021). Loss-of-function mutations typically reduce chloride conductance or impair channel trafficking to the plasma membrane, leading to diminish RPE ion transport capacity. Gain-of-function mutations, in contrast, can increase channel activity or alter gating behavior, potentially disrupting ionic transmission in RPE cells. Additional pathogenic mechanisms include dominant-negative effects, altered calcium sensitivity, and impaired protein stability.

Disruption of ionic homeostasis in RPE cells is thought to contribute directly to retinal degeneration by perturbing fluid balance, metabolic coupling, and photoreceptor support functions (Bok, 1993; Li Y. et al., 2017). These findings underscore the importance of precise functional characterization of *BEST1* variants and provide a mechanistic foundation for mutation-specific therapeutic strategies.

### 2.4 Disease spectrum and clinical implications

Clinically, bestrophinopathies exhibit marked phenotypic diversity, ranging from localized macular lesions to widespread retinal degeneration. This variability reflects both the functional heterogeneity of *BEST1* mutations and the complex interplay between ion transport, epithelial physiology, and retinal metabolism (Hartzell et al., 2005; Sparrow and Boulton, 2005; Bakall et al., 2007; Johnson et al., 2017; Sparrow and Kim, 2025).

The mechanistic link between BEST1 channel dysfunction and retinal degeneration underscores the importance of targeting epithelial transport pathways in therapeutic design. Rather than viewing bestrophinopathies solely as photoreceptor disorders, they can be conceptualized as primary diseases of RPE physiology with secondary photoreceptor involvement (Strauss, 2005; Davidson et al., 2009; Yang et al., 2015; Dalvin et al., 2016).

### 2.5 BEST1 as a paradigm for mechanism-guided retinal therapy

Studies of BEST1-associated retinopathies provide a paradigm for how mechanistic insight into ion channel regulation can inform therapeutic strategies. By establishing a direct connection between genetic mutation, channel dysfunction, and epithelial pathology, BEST1 research has enabled rational approaches to both gene-based and cell-based therapies (Yang et al., 2015; Zhao et al., 2021).

**FIGURE 1:**
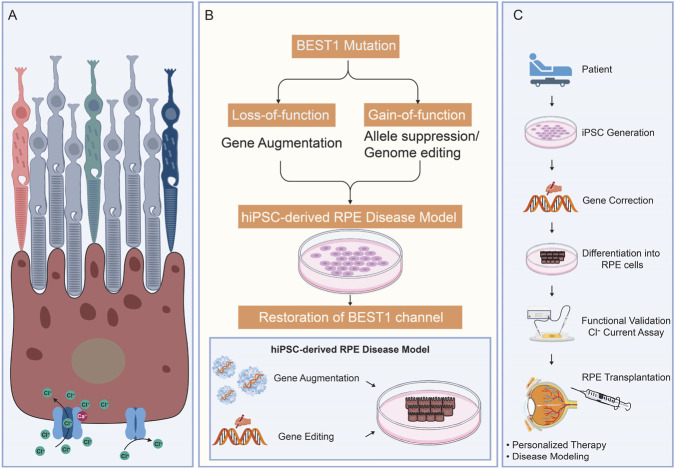
Integrated framework linking BEST1 dysfunction to regenerative therapeutic strategies. Schematic illustration of the physiological role of BEST1 in retinal pigment epithelium (RPE), the distinct pathogenic mechanisms associated with loss- and gain-of-function mutations, and corresponding therapeutic approaches. Human pluripotent stem cell–derived RPE (hPSC-RPE) models enable disease modeling and functional assessment of BEST1 variants. Gene-based strategies, including gene augmentation and genome editing, are tailored to specific mutation classes. Integration of gene correction with stem cell–derived RPE replacement represents a promising regenerative strategy for BEST1-associated retinopathies. **(A)** Best1 function in RPE cells. **(B)** Gene therapy strategies for BEST1 mutations. **(C)** Patient-specific RPE replacement strategy.

This framework illustrates how understanding the physiological role of a single ion channel can guide regenerative medicine at the tissue level. In this context, *BEST1* serves not only as a disease gene but also as a model for integrating molecular pathology with functional restoration in retinal repair strategies. These relationships between BEST1 channel dysfunction, mutation-specific mechanisms, and emerging therapeutic strategies are summarized in Figure 1.

## 3 Disease modeling using hiPSC-Derived RPE

Human induced pluripotent stem cell–derived retinal pigment epithelium (hiPSC-RPE) has emerged as a powerful platform for modeling inherited retinal diseases and evaluating therapeutic strategies. For BEST1-associated retinopathies, this approach addresses two major experimental limitations: the rarity of patient material and the limited accessibility of native human RPE cells. By generating RPE cells that carry defined *BEST1* mutations in a controlled *in vitro* environment, stem cell–based models provide a physiologically relevant system for dissecting disease mechanisms and testing corrective interventions (Li Y. et al., 2017; Kittredge et al., 2018b).

### 3.1 Establishment of hiPSC-derived RPE platforms

Protocols for differentiating human ESCs and iPSCs into RPE cells have enabled reproducible production of polarized epithelial monolayers expressing characteristic RPE markers and functional properties (Buchholz et al., 2009; Johnson et al., 2017; Li Y. et al., 2017; Kittredge et al., 2018b). These hiPSC-RPE cells exhibit typical morphological features, including pigmentation, tight junction formation, and apical–basolateral polarity, and they recapitulate key physiological functions of native RPE, such as transepithelial transport and ion channel activity.

The establishment of these systems has been particularly valuable for studying genes that are endogenously expressed in RPE, including *BEST1*. Because *BEST1* expression is largely restricted to this cell type in the retina, hiPSC-RPE models provide a uniquely appropriate context for evaluating the functional consequences of disease-associated mutations (Li Y. et al., 2017; Kittredge et al., 2021).

### 3.2 “Disease-in-a-dish” models for BEST1 dysfunction

The concept of “disease-in-a-dish” refers to the use of patient-specific stem cell–derived cells to recreate disease phenotypes *in vitro*. For BEST1-associated retinopathies, patient-derived iPSC-RPE cells carrying pathogenic mutations have been shown to reproduce key electrophysiological and molecular defects observed in affected individuals (Marmorstein et al., 2018; Ji et al., 2019b). These models enable direct correlation between genotype, channel function, and cellular physiology.

Using this approach, distinct *BEST1* mutations have been demonstrated to impair Ca^2+^-dependent Cl^−^ currents in RPE cells, thereby linking molecular defects in the BEST1 channel to altered epithelial ion transport (Zhang et al., 2010; Li Y. et al., 2017). Importantly, such phenotypes are measurable using functional assays, allowing quantitative comparison between different mutations and between corrected and uncorrected cells (Ji et al., 2019b; Zhao et al., 2021).

Beyond mechanistic insight, disease-in-a-dish systems provide a tractable platform for evaluating therapeutic strategies. Gene augmentation and genome editing approaches can be tested directly in patient-derived RPE cells, enabling assessment of functional rescue under near-physiological conditions (Li Y. et al., 2017; Zhao et al., 2021).

### 3.3 Modeling BEST1 mutations in a physiological context

Patient-derived iPSC-RPE models have been instrumental in demonstrating that BEST1 plays an indispensable role in mediating Ca^2+^-dependent Cl^−^ currents in human RPE cells (Li Y. et al., 2017; Kittredge et al., 2021). Functional analyses revealed that disease-associated mutations disrupt this current to varying degrees, providing a mechanistic link between molecular defects and impaired RPE physiology. Notably, electrophysiological measurements in iPSC-RPE cells showed close correspondence with patients’ clinical phenotypes, supporting the validity of this system as a translational disease model.

Subsequent studies expanded this approach to examine a broader spectrum of *BEST1* mutations, including both dominant and recessive variants (Ji et al., 2019b; Zhao et al., 2021). These investigations demonstrated that distinct mutations can produce heterogeneous functional outcomes, ranging from reduced channel activity to altered channel regulation. Importantly, the hiPSC-RPE platform enabled systematic comparison of mutation classes under controlled genetic and environmental conditions, revealing that pathogenic mechanisms are not uniform across bestrophinopathies. This insight has been critical for refining disease classification and for informing mutation-specific therapeutic strategies.

### 3.4 hiPSC-RPE models as a platform for therapeutic testing

Beyond disease modeling, hiPSC-RPE systems have served as a critical testing ground for therapeutic strategies targeting BEST1-associated retinopathies. Gene augmentation approaches using adeno-associated virus (AAV) vectors have been shown to restore Ca^2+^-dependent Cl^−^ currents in patient-derived RPE cells carrying loss-of-function mutations (Li Y. et al., 2017; Ji et al., 2019b). These proof-of-concept studies established that functional rescue at the cellular level is achievable and provided a rationale for mutation-specific gene therapy strategies.

More recently, hiPSC-RPE models have been used to evaluate combined therapeutic approaches for dominant or gain-of-function mutations, including gene supplementation together with suppression of endogenous mutant alleles (Zhao et al., 2021). Such studies underscore the value of patient-derived RPE cells as a preclinical platform for testing both conventional and emerging genome editing technologies. Importantly, functional restoration of ion channel activity serves as a quantifiable and disease-relevant biomarker, enabling objective assessment of therapeutic efficacy.

### 3.5 Advantages and limitations of hiPSC-RPE disease models

hiPSC-RPE systems offer several advantages over traditional heterologous expression models. They preserve endogenous gene regulation, native cellular architecture, and physiologically relevant signaling pathways, enabling more accurate interpretation of mutation-induced dysfunction (Kittredge et al., 2018b; Kittredge et al., 2021). In addition, patient-specific iPSC lines allow direct comparison of mutant and corrected isogenic controls, strengthening causal inference between genotype and phenotype (Zhao et al., 2021).

However, limitations remain. hiPSC-RPE cells may exhibit incomplete maturation compared with adult RPE, and inter-line variability can introduce experimental noise. Furthermore, these models lack the complex *in vivo* microenvironment of the retina, including interactions with photoreceptors, choroidal vasculature, and immune components (Wahlin et al., 2014). Despite these constraints, hiPSC-RPE systems represent a critical intermediate between molecular studies and animal models.

## 4 Gene therapy strategies for BEST1-associated retinopathies

The retina has long been considered an attractive target for gene therapy because of its relative immune privilege, well-defined cellular architecture, and accessibility for localized vector delivery (Yang et al., 2015; Guziewicz et al., 2016; Guziewicz et al., 2018). BEST1-associated retinopathies are particularly well suited for genetic intervention, as they arise from mutations in a single gene expressed predominantly in RPE cells (Carter et al., 2016; Li Y. et al., 2017). Advances in disease modeling using patient-derived RPE cells have provided a mechanistic foundation for the rational design of gene-based therapeutic strategies (Kittredge et al., 2018a; Kittredge et al., 2021). Figure 1 highlights how distinct mutation classes in *BEST1* necessitate different gene-based therapeutic strategies, including gene augmentation and genome editing.

### 4.1 Gene augmentation for loss-of-function mutations

Early studies demonstrated that many *BEST1* mutations lead to reduced or absent Ca^2+^-activated Cl^−^ currents in RPE cells, consistent with a loss-of-function phenotype (Li Y. et al., 2017; Ji et al., 2019b). In patient-specific iPSC-derived RPE models, supplementation with wild-type *BEST1* using AAV vectors restored Ca^2+^-dependent chloride currents and corrected functional defects at the cellular level (Li Y. et al., 2017; Ji et al., 2019b). These findings provided direct proof-of-concept that *BEST1* deficiency is amenable to gene augmentation therapy (Yang et al., 2015).

Importantly, both dominant and recessive loss-of-function mutations were shown to be rescuable by gene supplementation with comparable efficacy (Ji et al., 2019b; Sinha et al., 2020). This observation challenged the traditional assumption that dominant *BEST1* mutations necessarily act through gain-of-function mechanisms, suggesting that haploinsufficiency or dominant-negative effects may underlie many disease phenotypes (Ji et al., 2019b). From a translational perspective, these results support the feasibility of a relatively straightforward replacement strategy for a substantial subset of bestrophinopathies (Yang et al., 2015; Amato et al., 2023; Karaosmanoglu et al., 2024).

### 4.2 Distinct requirements for gain-of-function mutations

Subsequent work revealed that not all *BEST1* mutations respond similarly to gene augmentation. In particular, gain-of-function mutations exhibit strong dominant effects that cannot be overcome by simply increasing wild-type channel expression (Sinha et al., 2020; Zhao et al., 2021). In patient-derived RPE cells carrying such mutations, AAV-mediated delivery of wild-type *BEST1* failed to restore normal chloride currents, highlighting a fundamental mechanistic difference between mutation classes (Zhao et al., 2021).

These findings underscored the importance of mutation-specific therapeutic strategies. For gain-of-function variants, effective treatment requires not only supplementation of wild-type protein but also suppression of endogenous mutant alleles demonstrated that combining gene augmentation with CRISPR/Cas9-mediated knockdown of endogenous *BEST1* expression enabled functional rescue of Ca^2+^-activated Cl^−^ currents, providing a unified strategy capable of addressing both loss- and gain-of-function mutations (Zhao et al., 2021). This dual approach represents a conceptual advance in the treatment of dominant retinal channelopathies (Haldrup et al., 2025).

### 4.3 Genome editing and precision therapies

Beyond gene replacement, emerging genome editing technologies offer the possibility of correcting pathogenic *BEST1* mutations at their endogenous loci. CRISPR/Cas9-based editing has been explored in RPE cells as a means to selectively disrupt mutant alleles or restore normal gene sequence (Zhao et al., 2021). Base editing and prime editing further expand the toolkit for precise correction of single-nucleotide variants without introducing double-strand breaks.

hiPSC-derived RPE models provide a critical platform for evaluating these approaches under controlled conditions (Li Y. et al., 2017). By allowing direct measurement of endogenous channel function following genetic correction, these systems enable quantitative assessment of therapeutic efficacy and off-target effects (Ji et al., 2019b; Zhao et al., 2021). Moreover, functional restoration of Ca^2+^-dependent Cl^−^ currents serves as a disease-relevant biomarker, facilitating direct comparison among different editing strategies.

Despite their promise, genome editing approaches face several challenges, including delivery efficiency, potential off-target modifications, and long-term stability of corrected cells. Careful optimization of guide RNA design, editing efficiency, and RPE-specific targeting will be required before clinical translation can be realized (Yang et al., 2015).

### 4.4 Vector design and delivery considerations

AAV vectors remain the most widely used vehicles for retinal gene therapy due to their favorable safety profile and ability to transduce post-mitotic cells (Yang et al., 2015; Guziewicz et al., 2016; Scruggs et al., 2025). However, the packaging capacity of AAV imposes constraints on promoter selection and regulatory elements for *BEST1* expression. The use of RPE-specific promoters is critical to restrict expression to the appropriate cell type and minimize unintended effects in neighboring retinal layers (Esumi et al., 2009).

Delivery route is another important determinant of therapeutic outcome. Subretinal injection allows direct access to RPE cells but is surgically invasive, whereas intravitreal delivery offers a less invasive alternative with more limited RPE transduction efficiency (Yang et al., 2015; Guziewicz et al., 2016; Guziewicz et al., 2018). Advances in vector engineering and capsid modification may improve targeting and broaden the range of clinically feasible delivery strategies (Amato et al., 2023).

### 4.5 Translational challenges and future directions

Although substantial progress has been made in developing gene-based therapies for BEST1-associated retinopathies, several barriers remain. The heterogeneity of disease phenotypes, even among patients carrying the same mutation, complicates patient stratification and outcome prediction (Carter et al., 2016; Guziewicz et al., 2016). Long-term safety, immune responses, and durability of gene expression also require careful evaluation in preclinical and clinical studies (Amato et al., 2023).

Nevertheless, the convergence of stem cell–based disease modeling and gene therapy development has established BEST1 as a paradigm for mechanism-driven retinal therapeutics (Li Y. et al., 2017; Ji et al., 2019b; Zhao et al., 2021). Lessons learned from BEST1 provide a framework for extending similar strategies to other RPE-expressed ion channels and transporters implicated in retinal degeneration (Owji et al., 2021). As genome editing technologies mature and delivery platforms improve, precision therapies tailored to specific mutation classes are likely to become increasingly feasible.

Translational progress has also been demonstrated in naturally occurring canine models of bestrophinopathy. In these models, AAV-mediated BEST1 gene augmentation corrected the RPE-photoreceptor interface disease and reversed macro- and micro-detachments, providing strong preclinical support for therapeutic development (Guziewicz et al., 2018). This work was subsequently followed by an IND-enabling pharmacology/toxicity study of OPGx-BEST1 (AAV2-VMD2-BEST1) in canine models and helped support the launch of a Phase 1b/2a clinical trial for patients with BVMD or ARB (NCT07185256) (Choudhary et al., 2024).

## 5 Combining stem cell and gene therapy approaches

Stem cell–based retinal regeneration and gene-based therapeutic strategies have largely developed as parallel fields. However, increasing evidence suggests that their integration may offer synergistic advantages, particularly for monogenic retinal disorders such as BEST1-associated retinopathies (Yang et al., 2015; da Cruz et al., 2018). The convergence of these two approaches enables correction of disease-causing mutations at the cellular level followed by replacement of dysfunctional RPE, thereby addressing both genetic and cellular deficits (Schwartz et al., 2015; Mandai et al., 2017).

### 5.1 Gene correction at the stem cell stage

As illustrated in Figure 1, integration of gene correction with stem cell–derived RPE replacement represents a unified strategy to address both genetic and cellular defects. One promising strategy involves correcting pathogenic *BEST1* mutations at their endogenous loci using genome editing in patient-derived iPSCs, and corrected iPSCs can subsequently be differentiated into RPE cells that express functional BEST1 under native regulatory control (Soldner et al., 2011; Johnson et al., 2015; Maeder and Gersbach, 2016; Li Y. et al., 2017; Kittredge et al., 2018a; Anzalone et al., 2019).

This approach offers several conceptual advantages. First, genetic correction at the stem cell stage allows rigorous validation of genotype–phenotype relationships by comparison with uncorrected isogenic controls (Soldner et al., 2011; Ji et al., 2019b). Second, it minimizes the risk of dominant-negative or gain-of-function interference from mutant alleles, which has been shown to limit the efficacy of gene augmentation alone in certain BEST1 mutations (Zhao et al., 2021). Third, autologous transplantation of corrected iPSC- RPE cells has the potential to reduce immune rejection and obviate the need for long-term immunosuppression (Mandai et al., 2017; da Cruz et al., 2018).

### 5.2 Functional assessment and long-term integration

A critical challenge in combined stem cell and gene therapy strategies is demonstrating durable functional integration of transplanted cells. Beyond morphological engraftment, corrected RPE cells must establish appropriate polarity, tight junctions, and interactions with photoreceptors (Mandai et al., 2017; Kittredge et al., 2018b). Functional assays measuring transepithelial transport, Ca^2+^-dependent Cl^−^ currents, and visual cycle–related processes provide objective benchmarks for evaluating therapeutic success (Li Y. et al., 2017; Ji et al., 2019b).

Animal models offer an essential intermediate platform for assessing long-term survival, safety, and physiological integration of corrected RPE cells (Mandai et al., 2017; da Cruz et al., 2018). Importantly, functional restoration of BEST1-dependent ion channel activity may serve as a sensitive biomarker for therapeutic efficacy, linking molecular correction to tissue-level recovery (Li Y. et al., 2017; Zhao et al., 2021). Such quantitative endpoints are particularly valuable for optimizing transplantation protocols and comparing alternative editing strategies (Soldner et al., 2011; Maeder and Gersbach, 2016).

### 5.3 Lessons from BEST1 for retinal regeneration

Studies of BEST1 provide a broader conceptual framework for retinal regeneration. They demonstrate that restoration of a single ion channel can exert disproportionate effects on epithelial physiology and photoreceptor maintenance (Li Y. et al., 2017; Ji et al., 2019b). This highlights the importance of targeting not only photoreceptors but also the supporting RPE layer in regenerative strategies (Schwartz et al., 2015; Yang et al., 2015).

Moreover, the BEST1 paradigm illustrates how mechanistic understanding of ion channel function can guide regenerative medicine. Rather than relying solely on structural replacement, integrating functional correction with cell replacement ensures that regenerated tissues possess appropriate physiological properties. This principle may be extended to other retinal disorders involving epithelial transporters, channels, or metabolic regulators (da Cruz et al., 2018; Battu et al., 2022; Maeda et al., 2022; Sanie-Jahromi and Nowroozzadeh, 2022).

### 5.4 Toward integrated regenerative therapies

The integration of stem cell technology with gene correction strategies represents a shift toward comprehensive regenerative medicine for inherited retinal diseases. In this model, disease-specific mutations are corrected *in vitro*, differentiated into therapeutically competent RPE cells, and transplanted to restore retinal homeostasis (Mandai et al., 2017; Zhao et al., 2021). Although substantial technical challenges remain—including large-scale cell production, surgical delivery, and long-term safety monitoring—the conceptual foundation has been firmly established by advances in BEST1 research (Yang et al., 2015; Li Y. et al., 2017).

As genome editing and stem cell differentiation protocols continue to mature, combined approaches are likely to play an increasingly prominent role in retinal regenerative medicine (Maeder and Gersbach, 2016; Anzalone et al., 2019). BEST1-associated retinopathies thus serve not only as a target for therapy but also as a prototype for integrating molecular correction with tissue-level regeneration.

## 6 Broader implications for retinal regeneration

Studies of BEST1-associated retinopathies provide a broader conceptual framework for retinal regeneration that extends beyond a single gene or disease (Figure 1). They illustrate how disruption of a specific ion channel can profoundly alter RPE physiology and secondarily compromise photoreceptor survival. This paradigm emphasizes that effective retinal regeneration requires restoration not only of cellular structure but also of essential physiological functions that support long-term tissue homeostasis (Hartzell et al., 2005; Strauss, 2005).

### 6.1 Beyond BEST1: ion channels and transporters as regulators of retinal homeostasis

Although BEST1 represents one of the most extensively characterized RPE ion channels, it is increasingly evident that multiple ion channels and transporters cooperate to maintain retinal ionic and metabolic balance, including other CaCCs like members of the anoctamin (TMEM16) family (Hartzell et al., 2005; Strauss, 2005; Yang et al., 2014a; Yang and Colecraft, 2016). Dysregulation of these pathways can perturb fluid transport, oxidative balance, and metabolic coupling between RPE and photoreceptors (Marmorstein and Kinnick, 2007).

This suggests that retinal degeneration may, in some cases, arise from failures in epithelial transport and ionic homeostasis rather than primary defects in phototransduction alone. Accordingly, regenerative strategies that focus exclusively on photoreceptor replacement may be insufficient unless the underlying epithelial support system is also restored (Strauss, 2005; Yang et al., 2015). The BEST1 paradigm highlights the importance of targeting epithelial ion channel function as a complementary axis of retinal regeneration.

### 6.2 Stem cell–based retinal tissue engineering

Advances in stem cell biology have enabled the generation of increasingly complex retinal tissues, including three-dimensional retinal organoids and RPE–photoreceptor co-culture systems (Eiraku and Sasai, 2012; Zhong et al., 2014). These platforms provide opportunities to examine how ion transport, metabolic exchange, and epithelial polarity contribute to tissue-level function (Buchholz et al., 2009; Wahlin et al., 2014). Incorporating mechanistic insights from BEST1 and related channels into such models may improve their physiological relevance and predictive power for therapeutic development (Li M. H. et al., 2017; Kittredge et al., 2018b).

Moreover, tissue engineering approaches that combine RPE replacement with photoreceptor support may benefit from ensuring that regenerated epithelial layers express properly regulated ion channels and transporters. Functional maturation of transplanted cells, rather than morphological integration alone, is likely to be a key determinant of long-term success in regenerative therapies (Schwartz et al., 2015; da Cruz et al., 2018).

### 6.3 From single-gene repair to functional regeneration

The BEST1 disease model illustrates a transition from gene-centered repair strategies toward a more integrated view of tissue regeneration. Restoration of BEST1-dependent chloride currents in patient-derived RPE cells has been shown to correlate with recovery of epithelial function, supporting the concept that ion channel activity can serve as a functional biomarker of therapeutic efficacy (Li M. H. et al., 2017; Ji et al., 2019b; Zhao et al., 2021). This linkage between molecular correction and physiological outcome provides a quantitative framework for evaluating regenerative interventions.

More broadly, this approach supports a shift from correcting isolated genetic lesions to re-establishing coherent physiological networks within regenerated tissue. Ion channels, transporters, and metabolic regulators form interconnected systems that define epithelial identity and function. Regenerative strategies that incorporate these functional parameters may achieve greater durability and clinical relevance than those focused solely on cellular replacement (Yang et al., 2015; Mandai et al., 2017).

### 6.4 Implications for personalized retinal therapies

The diversity of *BEST1* mutations and their distinct functional consequences underscore the need for personalized therapeutic strategies. Stem cell–derived disease models enable stratification of mutations based on functional phenotype, facilitating tailored intervention strategies such as gene augmentation, allele-specific suppression, or precise genome editing (Bassuk et al., 2016; Ji et al., 2019b; Zhao et al., 2021). This precision-medicine framework may be extendable to other inherited retinal disorders involving epithelial dysfunction (Wahlin et al., 2014).

Importantly, the ability to evaluate therapeutic correction *in vitro* using patient-specific cells establishes a feedback loop between mechanistic insight and translational design (Li M. H. et al., 2017; Kittredge et al., 2018b). Such iterative refinement is likely to be critical for optimizing regenerative therapies in genetically heterogeneous diseases.

### 6.5 Toward integrated retinal regenerative medicine

Collectively, research on BEST1-associated retinopathies demonstrates how mechanistic understanding of ion channel function can guide the development of regenerative strategies at the tissue level (Hartzell et al., 2005; Yang et al., 2015). Rather than treating gene therapy and cell therapy as independent modalities, integration of molecular correction with cellular replacement offers a unified framework for addressing both genetic and physiological deficits.

This integrated paradigm may represent a generalizable model for retinal regeneration: disease-causing mutations are corrected *in vitro*, differentiated into functionally competent epithelial cells, and transplanted to restore ionic homeostasis and photoreceptor support (Schwartz et al., 2015; Mandai et al., 2017; da Cruz et al., 2018). While substantial challenges remain, including scalability, surgical delivery, and long-term safety, the conceptual foundation established by BEST1 research provides a roadmap for future regenerative interventions (West et al., 2020; Sharma and Jaganathan, 2021; Maeda et al., 2022).

## 7 Conclusion and future perspectives

Research on BEST1-associated retinopathies has established a direct link between ion channel dysfunction in the RPE and retinal degeneration. These studies demonstrate that disruption of CaCC is sufficient to compromise epithelial homeostasis and, in turn, photoreceptor viability (Strauss, 2005; Li Y. et al., 2017). Beyond its disease relevance, BEST1 has emerged as a model system illustrating how precise regulation of epithelial ion channels is integral to retinal physiology and visual function (Marmorstein and Kinnick, 2007; Zhao et al., 2021).

Over the past decade, advances in iPSC technology and genome engineering have transformed the experimental and therapeutic landscape for inherited retinal disorders. hiPSC–derived RPE models have enabled direct investigation of disease mechanisms in patient-specific genetic contexts (Kittredge et al., 2018b; Ji et al., 2019b), while gene-based strategies have provided proof-of-concept for functional rescue of *BEST1* mutations (Yang et al., 2015; Li Y. et al., 2017; Zhao et al., 2021). Together, these developments highlight the complementary strengths of stem cell–based regeneration and molecular correction approaches.

Despite this progress, substantial challenges remain before integrated regenerative therapies can be widely implemented. Genome editing strategies must achieve high precision and safety, particularly in the context of dominant or gain-of-function mutations (Komor et al., 2016; Zhao et al., 2021) Stem cell–derived RPE must reach appropriate levels of functional maturity and demonstrate stable long-term integration after transplantation (Mandai et al., 2017; da Cruz et al., 2018). In addition, issues related to immune compatibility, scalable cell production, and surgical delivery continue to require systematic optimization (Tsai and Joung, 2016; Sharma et al., 2020).

An important conceptual lesson from BEST1 research is that successful retinal regeneration will likely require restoration of both cellular architecture and physiological function. Replacement of diseased cells alone may be insufficient if critical ion transport processes are not properly re-established. Conversely, molecular correction without durable cellular replacement may fail to address progressive epithelial loss. Thus, future therapies are expected to rely increasingly on strategies that integrate genetic correction with tissue-level regeneration (Yang et al., 2015; Mandai et al., 2017; Zhao et al., 2021).

Looking forward, BEST1-associated retinopathies provide a valuable prototype for mechanism-guided regenerative medicine. By linking defined molecular defects to epithelial dysfunction and clinical pathology, this system illustrates how basic ion channel biology can inform therapeutic design (Strauss, 2005; Li M. H. et al., 2017). As stem cell differentiation methods and gene editing technologies continue to mature, similar integrative frameworks may be extended to other retinal disorders involving transporters, metabolic enzymes, or structural proteins (Gonzalez-Cordero et al., 2017).

In summary, studies of BEST1 have advanced understanding of retinal epithelial physiology while simultaneously shaping emerging strategies for retinal repair. The convergence of mechanistic insight, stem cell biology, and gene therapy offers a rational path toward durable treatment of inherited retinal diseases. Continued progress will depend on close integration of molecular research with translational and clinical efforts, ultimately defining the next phase of retinal regenerative medicine (Yang et al., 2015; Mandai et al., 2017; Zhao et al., 2021).
